# How AI competencies can make B2B marketing smarter: strategies to boost customer lifetime value

**DOI:** 10.3389/frai.2024.1451228

**Published:** 2024-12-18

**Authors:** Tayyeba Bashir, Tan Zhongfu, Burhan Sadiq, Ammara Naseem

**Affiliations:** ^1^School of Economics and Management, North China Electric Power University, Beijing, China; ^2^College of Economics and Management, Beijing University of Technology, Beijing, China

**Keywords:** artificial intelligence competencies, B2B marketing capabilities, customer lifetime value, information management, resource-based view theory

## Abstract

There has been a rapid rise in utilization of artificial intelligence (AI) in many different sectors in the last several years. However, business-to-business (B2B) marketing is one of the more notable examples. The initial assessments emphasize the significant advantages of AI in B2B marketing, including its knack for yielding unique understandings into consumer behaviors, recognizing crucial market trends, and improving operational efficiency. However, there seems to be a limited grasp of the optimal way to develop artificial intelligence competencies (AIC) for B2B marketing and how these attributes inevitably affect customer lifetime value (CLV). Equipped with AIC and B2B marketing literary fiction, this research unveils a theoretical research framework for evaluating the repercussions of AIC on B2B marketing capabilities and, subsequently, on CLV. We analyze the suggested research model using partial least squares structural equation modeling (PLS-SEM), leveraging 367 survey replies from Pakistani companies. The outcomes show a significant relationship that describe the ability to leverage AIC to enhance CLV, and also signifies the mediating role of B2B marketing capabilities to enhance CLV by integrating AIC in internet marketing. The findings of this study provide practical implications for marketers to monetize their marketing skills to enhance CLV and researchers with theoretical underpinnings of integration of AIC into marketing.

## 1 Introduction

The integration of AI into B2B marketing processes has gained substantial attention in recent years due to its potential to significantly enhance marketing capabilities and drive customer value. AI skills like infrastructure, business spanning, and being proactive have become important for making data-driven decisions, personalizing marketing, and using predictive analytics in B2B settings (Dwivedi et al., 2021). Despite these advancements, there is still a limited understanding of how to systematically leverage AIC to enhance B2B marketing capabilities, especially in emerging economies like Pakistan. In Pakistan, where businesses are navigating the digital landscape, AI adoption remains fragmented, especially within B2B sectors. Companies often struggle with the strategic implementation of AI, which hinders their ability to maximize CLV through improved marketing performance (Moradi and Dass, 2022). This study addresses this gap by exploring how AIC can enhance B2B marketing processes and subsequently improve CLV. The core problem driving this research is the misalignment between technological advancements in AI and their practical application in B2B marketing to achieve sustainable customer relationships and value maximization (Dwivedi, 2024).

Existing literature provides insights into AI’s transformative potential in various business domains, but few studies have explicitly examined the relationship between AIC and B2B marketing, with CLV as the focal outcome (Tripathi et al., 2021; AboElHamd et al., 2020). Most research has focused on the general adoption of AI in marketing but has not thoroughly investigated its role in enhancing marketing information management, planning, and implementation in B2B contexts. Furthermore, we have yet to fully explore the mediating role of B2B marketing processes in the relationship between AIC and CLV. This gap leads to several pertinent research questions: How do AI infrastructure, business spanning, and proactive stance directly impact B2B marketing capabilities? What role do marketing processes play in mediating the relationship between AIC and CLV? How does the improvement in B2B marketing activities affect CLV? This study aims to provide both theoretical and practical insights into how AI can optimize marketing performance and customer value creation in B2B markets, guided by these questions (Saura et al., 2021).

Drawing upon these research questions, the main objective of this research is to examine the relationship between AIC and B2B marketing processes and how these processes mediate the impact on CLV. The study addresses the identified research gap by using quantitative empirical analysis, specifically PLS-SEM, to evaluate data collected from 367 Pakistani companies. It is possible to look into the complicated connections between AI infrastructure, business spanning, and a proactive stance, along with marketing tasks like information management, planning, and implementation (Ebaietaka, 2024), using the PLS-SEM method. By systematically examining these relationships, this research contributes to the literature on AI-driven marketing by identifying how AI can enhance B2B marketing outcomes. Additionally, the study has significant practical implications, offering strategies for businesses to align AIC with marketing functions to maximize CLV. This research contributes to both theory and practice by providing a comprehensive framework that connects AIC with improved marketing and customer outcomes (Curiskis et al., 2023). The findings are particularly relevant for emerging markets, where the integration of AI into B2B marketing is still in its early stages but holds considerable potential.

This research paper’s structure ensures a logical and coherent presentation of the study’s objectives, methodology, and findings. The next section after this introduction presents a comprehensive literature review, which explores the theoretical foundations of AIC, B2B marketing, and CLV. The second section discusses the methodology used in this study, including data collection, sampling, and the statistical techniques employed. The third section presents the results of the empirical analysis with a detailed discussion of the relationships between AIC, B2B marketing, and CLV. Finally, the paper concludes with managerial implications, limitations of the study, and suggestions for future research.

## 2 Literature review

### 2.1 Application of resource-based view theory

The Resource-Based View (RBV) theory provides a strong theoretical foundation for understanding how firms can leverage internal resources to achieve competitive advantage and improve performance outcomes. According to RBV, resources that are valuable, rare, inimitable, and non-substitutable (VRIN) provide firms with a sustained competitive advantage (Barney, 1991). This study identifies AIC, such as infrastructure, business spanning, and a proactive stance, as critical organizational resources that can enhance marketing capabilities and ultimately drive CLV. These AIC, when aligned with strategic marketing functions, serve as rare and valuable resources that can facilitate improved decision-making, personalized customer engagement, and enhanced data analytics, all of which are critical to maintaining long-term customer relationships (Teece et al., 2016). The RBV framework thus forms the basis for investigating how AIC can enable firms to outperform competitors in B2B marketing by enhancing their ability to manage information, plan strategically, and implement marketing initiatives effectively.

The application of RBV in AI-driven B2B marketing is particularly relevant because AI technologies allow firms to exploit their existing resources while simultaneously building new capabilities. AI infrastructure, for instance, represents the technological backbone that enables firms to collect, analyze, and leverage large datasets, offering insights that were previously inaccessible (Jarrahi, 2018). This infrastructure not only supports marketing processes but also allows businesses to gain a deeper understanding of customer behavior and preferences, thereby enhancing customer engagement and value creation (Liu et al., 2021). Similarly, the concept of business spanning in AI capabilities refers to the firm’s ability to integrate AI across multiple functional areas, fostering collaboration and improving efficiency in B2B marketing activities. By spanning business functions, firms can enhance information management, optimize marketing planning, and ensure effective implementation of strategies, thus making AI an indispensable resource for driving CLV (Laksono et al., 2023). A proactive stance, as another key AI capability, reflects the firm’s commitment to anticipating market trends and customer needs. Firms that adopt a proactive AI strategy are better positioned to respond to market dynamics and create personalized marketing experiences that resonate with B2B customers, further solidifying long-term relationships (Alsharafa et al., 2024).

### 2.2 Review of existing literature

The literature on AI in marketing has grown substantially in recent years, highlighting the transformative potential of various AI technologies and methodologies, such as machine learning, natural language processing, and quality function deployment (QFD) in enhancing marketing performance. Research by Sben et al. (2024) emphasizes how AI infrastructure integrated with various AI technologies and methodologies can streamline information management, allowing firms to gather, analyze, and act on customer data more efficiently than traditional methods. Information management plays a critical role in B2B marketing, where firms must handle complex and often large-scale customer relationships. QFD, for example, is particularly valuable as it structures AI applications around customer requirements and expert-driven processes, thus improving marketing precision and outcomes with customer needs and quality standards (Firmansyah et al., 2024). Additionally, various AI methodologies such as automated content generation, recommendation algorithms, and predictive analytics enhance marketing planning by allowing firms to engage in predictive modeling and scenario analysis, which help in developing more effective and data-driven marketing strategies (Li et al., 2021). The integration of QFD with AI in marketing provides a structured framework for incorporating expert judgment into data-driven decision-making, enhancing the relevance and precision of marketing actions (Rane et al., 2024).

Despite the growing body of research on AI in marketing, there is still a significant gap in understanding the mediating role of B2B marketing processes in the relationship between AIC and CLV. While many studies acknowledge the importance of AI in driving marketing outcomes, few have explicitly examined how AIC translates into enhanced marketing performance through the mediating role of specific marketing processes (Kumari et al., 2024). The current research proposes that AI capabilities spanning data analysis, customer personalization, and cross-functional integration influence CLV by enhancing the efficiency and effectiveness of B2B marketing processes. Sun et al. (2023) say that companies gain a competitive edge by using their own resources (in this case, AIC) to improve their operational capabilities (B2B marketing processes), which in turn lead to better performance outcomes (CLV). Furthermore, this research, which focuses on the specific context of Pakistani firms, offers valuable insights into leveraging these AI methodologies in emerging markets, where the adoption of such technologies and methods is still in its nascent stages (Alkobaisi and Al, 2024).

Research has demonstrated that firms with strong AI infrastructure, including data-driven customer insight generation and cross-departmental integrations, are better equipped to collect and analyze large datasets, leading to more informed marketing strategies and improved operational efficiency (Bag et al., 2022). By enhancing information management, firms can gain deeper insights into customer behavior and preferences, enabling more effective marketing planning and implementation (Shin and Kang, 2022). The integration of AI across multiple functional areas within the firm ensures that marketing teams work collaboratively with sales, customer service, and other departments, leading to more cohesive and effective marketing strategies (Liu et al., 2020). By leveraging specific AI-driven methods and technologies, such as QFD for customer-centric product planning and natural language processing for real-time customer feedback, firms can optimize their marketing processes, improving customer engagement and driving value creation (Jorzik et al., 2023). Research has shown that firms with advanced AIC are better able to deliver personalized marketing experiences, optimize customer interactions, and foster long-term relationships, all of which contribute to higher CLV (Paschen et al., 2020). By leveraging these AI techniques to enhance marketing processes, firms can increase customer satisfaction, loyalty, and retention, leading to sustained customer value over time (Bag et al., 2021).

**Figure 1:**
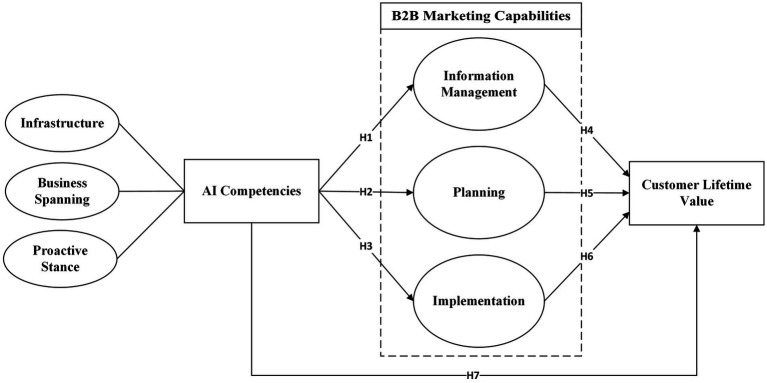
Conceptual framework.

Thus, this study’s conceptual framework (Figure 1) is based on the idea that AIC capabilities can enhance B2B marketing processes, thereby mediating the impact of AI technologies and methodologies on CLV. Previous studies (Ebaietaka, 2024) support this framework by highlighting the role of AI in enhancing marketing efficiency, personalization, and customer engagement. However, unlike previous research that often examines the direct relationship between AI and marketing outcomes, this study emphasizes the mediating role of marketing processes, providing a more nuanced understanding of how AI approaches, specific technologies like QFD can enhance customer value in B2B contexts. This study makes a contribution to both theory and practice by looking into how AIC, B2B marketing processes, and CLV work together. It does this by providing a complete model that can help companies use AI and its various methods to improve their B2B marketing.

## 3 Methodology

### 3.1 Data, surveys, and administration

The data collection process for this study followed a structured and systematic approach to ensure the validity and reliability of the results. The research employed a primary data collection method, gathering responses directly from 367 Pakistani companies operating in the B2B sector. These companies were selected based on their adoption of AI technologies within their marketing processes. The choice of Pakistani firms provided a unique perspective on the adoption and impact of AI in emerging markets, where technological infrastructure and resources might differ from more developed regions (Lee and Mangalaraj, 2022).

To ensure a representative sample, the survey was distributed through a combination of email invitations and online survey platforms. Convenience sampling was utilized, targeting senior managers, marketing executives, and IT professionals within these companies, as they are most likely to possess insights into the firm’s AIC and marketing strategies. Prior to distributing the survey, a pilot test was conducted with a small group of respondents to assess the clarity and relevance of the items. Based on feedback, minor revisions were made to the wording of some questions to enhance comprehension. The sample size exceeded the recommended threshold for PLS-SEM, which typically requires a minimum sample size of 100–200 observations depending on the complexity of the model (Hair Jr et al., 2020). As the study involves several latent variables with multiple indicators, the sample size of 367 provided adequate statistical power to detect meaningful relationships between variables. The process of data collection was conducted from September 2023 to January 2024, yielding a total of 367 completed responses Table 1.

**Table 1:** Sample and respondents for descriptive statistics.

Factors	Percentage (%)
Sectors	
Media	8.2%
Transport	3.7%
Telecommunications	19.3%
Bank and Financials	4.8%
Oil and Gas	6.2%
Consumer Goods	10.2%
Industrials (Industrial/Construction Goods)	8.2%
Others (Basic Materials, Consumer Services, Shipping, etc.)	29.7%
Technology	9.7%
Size of the Firm (in terms of employees)	
1–10	12.5%
11–50	26.9%
51–200	32.8%
201 +	27.8%
Number of Years Using AI	
< 1 Year	10.2%
1–2 Years	22.2%
2–3 Years	27.1%
3–4 Years	25.4%
4 + Years	15.1%
Position of the Respondent	
Presidents /CEO	6.2%
Chief Information Officer	15.6%
Senior Vice President	6.4%
Executive & Executive Assistant	10.4%
Manager & Assistant Manager	35.8%
Administrative Assistant	25.6%

The majority of them belonged to the telecommunication sector, the industrial sector, the media sector, and the consumer product sectors. Moreover, the majority of the enterprises possessed prior experience with AI in their business operations, with most having a minimum of 2–3 years of experience. Additionally, a large number of the sample had been associated with AI for greater than 3–4 years in the companies they worked for. The responders were highly suitable for the questions we asked, as most of them were administrative assistants and assistant managers with expertise in their firms’ IT and business domains.

### 3.2 Measures

We developed a survey instrument as the primary data collection tool to capture respondents’ perceptions of AIC, B2B marketing processes, and CLV. We structured the survey into four main sections: AI infrastructure, business spanning, proactive stance, B2B marketing processes (information management, planning, and implementation), and CLV. We populated each section with items adapted from validated scales in prior literature to ensure the reliability of the constructs. For instance, we adapted measures of AI infrastructure and capabilities from previous studies that examined the adoption of AI technologies in marketing (Kumar et al., 1993; Mikalef et al., 2023). We assessed B2B marketing processes using scales from (Kumar et al., 1993) related to information management, marketing planning, and implementation, and measured CLV using established scales from relationship marketing literature (Firmansyah et al., 2024). Details of the questionnaire is presented in Appendix 1.

Respondents rated the survey items on a 5-point Likert scale, which ranged from “strongly disagree” to “strongly agree,” expressing their level of agreement with each statement. This approach provided a standardized method for measuring perceptions across different companies, enabling the collection of comparable data for subsequent statistical analysis.

### 3.3 Ethical considerations

We conducted this study in accordance with the ethical standards of the institutional and/or national research committee, the 1964 Helsinki Declaration and its later amendments, or comparable ethical standards. We obtained informed consent from all individual participants included in the study. We fully informed the participants about the purpose, procedures, potential risks, and their rights, including the right to withdraw from the study at any time. We documented and securely stored the written consent.

## 4 Data analysis

PLS-SEM, a robust technique suitable for testing complex relationships among latent variables in empirical research, conducted the data analysis for this study. We chose PLS-SEM for its ability to handle small to medium sample sizes and its suitability for exploratory studies, where the primary goal is to explain variance in dependent variables and assess predictive relevance (Hair Jr et al., 2016). We performed the analysis in this research using Smart PLS 4, a widely recognized software tool for PLS-SEM. We conducted the analysis in two stages: measurement model assessment and structural model evaluation.

### 4.1 Measurements model evaluation

We evaluated the reliability and validity of the constructs in the measurement model assessment to ensure the consistency and accuracy of the indicators used to measure latent variables. The first step involved examining internal consistency reliability using Cronbach’s alpha (Ca) and composite reliability (CR). Both of these indicators needed to exceed the threshold of 0.70, ensuring that the items within each construct consistently measure the same underlying concept (Hair Jr et al., 2016). All constructs in this study demonstrated adequate internal consistency, with Ca and CR values exceeding 0.70. Next, we assessed convergent validity using Average Variance Extracted (AVE), a method that gages the degree of correlation between a construct’s indicators (Fornell and Larcker, 1981a). The AVE values for all constructs exceeded this threshold, confirming that the indicators are adequately representative of the underlying latent variables. The study looked at the measurement and structural models as well as the possibility of multicollinearity between the independent variables by finding the variance inflation factor (VIF). In this analysis, the VIF values for all independent variables were well below the threshold of 5, with most values ranging between 1.2 and 2.3. These values suggest that this model does not worry about multicollinearity, allowing for confident interpretation of the relationships between the independent variables, dependent variables, and mediating variables. Table 2 below illustrates the results.

**Table 2:** Measurement model evaluation.

	Outer loadings	VIF	Cα	CR	AVE
BSAP1	1.024	2.055	0.835	0.924	0.858
BSAP2	0.977	2.055			
CLV1	0.845	1.588	0.826	0.884	0.655
CLV2	1.138	2.300			
CLV3	1.209	2.328			
CLV4	0.937	1.560			
IMPL1	1.026	2.421	0.869	0.920	0.793
IMPL2	1.024	2.926			
IMPL3	0.954	2.006			
INFM1	1.026	1.858	0.838	0.891	0.672
INFM2	0.989	2.331			
INFM3	0.995	1.993			
INFM4	0.985	1.699			
INFS1	1.007	1.629	0.766	0.895	0.810
INFS2	0.994	1.629			
PLAN1	0.973	1.789	0.863	0.907	0.709
PLAN2	0.901	1.911			
PLAN3	1.078	2.348			
PLAN4	1.049	2.444			
PROS1	0.91	1.774	0.845	0.896	0.683
PROS2	1.044	1.826			
PROS3	1.061	2.851			
PROS4	1.016	2.779			

We then evaluated discriminant validity using two approaches: the Fornell-Larcker criterion and the Heterotrait-Monotrait (HTMT) ratio of correlations. The Fornell-Larcker criterion checks whether the square root of the AVE for each construct is greater than its correlation with other constructs, ensuring that each construct is distinct from others (Fornell and Larcker, 1981b). We also examined the HTMT ratio to further confirm discriminant validity, with values below 0.85 indicating that the constructs are not excessively similar. Both criteria were satisfied in this analysis, confirming that the constructs in the model were sufficiently distinct, as shown in Table 3.

**Table 3:** Discriminant validity.

	BSAP	CLV	IMPL	INFM	INFS	PLAN	PROS
Fornell-Lacker Criteria							
BSAP	0.926						
CLV	0.445	0.810					
IMPL	0.320	0.444	0.890				
INFM	0.340	0.369	0.211	0.820			
INFS	0.613	0.369	0.267	0.272	0.900		
PLAN	0.429	0.448	0.557	0.295	0.469	0.842	
PROS	0.419	0.340	0.323	0.204	0.381	0.407	0.827
HTMT-Ratio							
BSAP	0.526						
CLV	0.375	0.517					
IMPL	0.398	0.426	0.246				
INFM	0.766	0.456	0.328	0.333			
INFS	0.506	0.518	0.644	0.347	0.573		
PLAN	0.493	0.399	0.372	0.232	0.468	0.476	
PROS							

To make sure that the higher-order construct of AIC was valid and reliable, we first looked at how the formative lower-order constructs affected their higher-order counterparts. These were three first-order constructs. We found all weights to be positive and significant on the entrusted higher-order construct. Next, we needed to assess whether the initial order constructs showed any signs of multicollinearity. So as to investigate this further, we conducted calculations on the VIF values and established a cut-off threshold of 3.3 (Henseler et al., 2015). All of the values of first-order constructs were less than the threshold, indicating that multicollinearity was not a concern in our sample, as shown in Table 4.

**Table 4:** Validation of higher order constructs.

Construct	Measures	Weight	Significance	VIF
AIC	INFS	0.411	*p* < 0.001	1.652
	BSAP	0.449	*p* < 0.001	1.711
	PROS	0.378	*p* < 0.001	1.250

### 4.2 Structural model evaluation

We conducted a PLS-SEM analysis to determine the path coefficients once we confirmed the properties of the measurement model. We assessed the structural model using its path coefficients, coefficient of determination $\left(R^{2}\right)$ , effect size $\left(f^{2}\right)$ , and predictive relevance $\left(Q^{2}\right)$ . The R^2^ values for B2B marketing processes and CLV showed a high level of explanatory power. This is especially true in marketing research, where R^2^ values between 0.50 and 0.75 are thought to be moderate to high (Hult et al., 2018). The $\left(Q^{2}\right)$ values for both B2B marketing processes and CLV were above zero, confirming that the model has sufficient predictive relevance for these constructs. A $\left(Q^{2}\right)$ value greater than zero indicates that the model can accurately predict the outcomes for the dependent variables, which is crucial for validating the model’s practical implications (Shmueli et al., 2019). Table 5 provides the results.

**Table 5:** Effect size, R-square & Q-square.

	F-square				R-square	Q^2^
	Customer lifetime value	Planning	Information management	Implementation	Endogenous constructs	
AI Competencies	0.070	0.410	0.132	0.165		
Planning	0.012				0.291	0.282
Information Management	0.052				0.117	0.107
Implementation	0.061				0.141	0.132
Customer Lifetime Value					0.356	0.222

**Figure 2:**
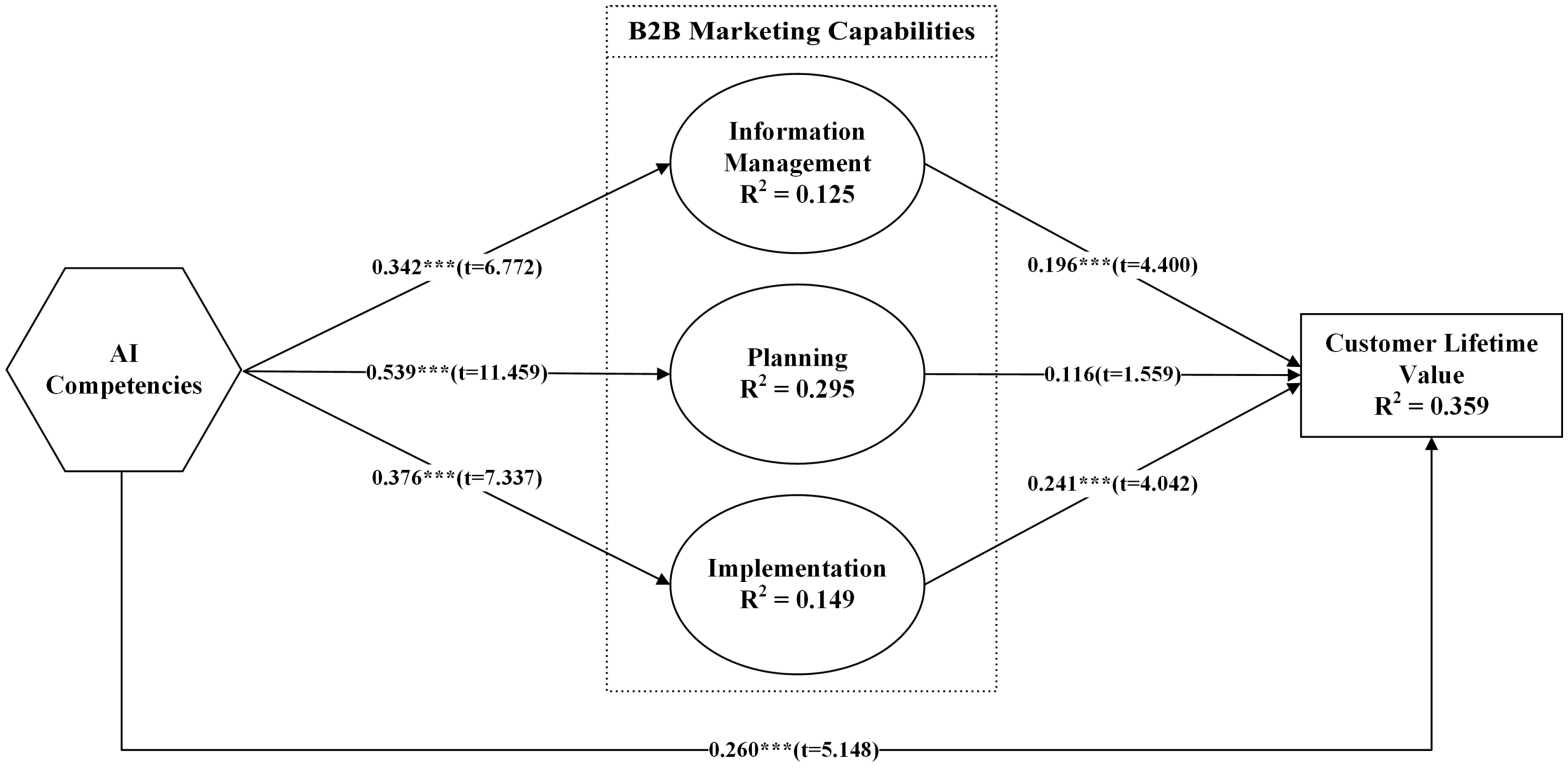
Structural model evaluation results.

Figure 2 and Table 6 specifically display the analysis’s findings. We examined the path coefficients to test the strength and significance of the relationships between variables. We determined the significance of these relationships using a bootstrapping technique with 5,000 subsamples, which generates t-values and *p*-values to test the hypotheses (Zhao et al., 2010). After conducting an in-depth analysis, it is evident that AIC has a positive and significant effect on all three B2B marketing capabilities. It is evident that AIC strongly influences INFM, resulting in a more significant and discernible effect (*β* = 0.342, *t* = 6.772, *p* < 0.000). Also, AIC had a significant and positive effect on PLAN (*β* = 0.047, *t* = 11.459, *p* < 0.000) and IMPL (*β* = 0.376, *t* = 7.337, *p* < 0.000). It has been observed that effective INFM has a significant and positive impact on CLV (*β* = 0.196, *t* = 4.400, *p* < 0.000), as well as on IMPL (*β* = 0.241, *t* = 4.042, *p* < 0.000). However, the PLAN did not have a significant impact on CLV (*β* = 0.116, *t* = 1.559, *p* = 0.119).

**Table 6:** Results of direct relationships.

Hypothesis	Structural path	Effect	*t*-value	Conclusion
H1	AIC → INFM	0.342	6.772***	Supported
H2	AIC → PLAN	0.539	11.459***	Supported
H3	AIC → IMPL	0.376	7.337***	Supported
H4	INFM → CLV	0.196	4.400***	Supported
H5	PLAN → CLV	0.116	1.559	Not-Supported
H6	IMPL → CLV	0.241	4.042***	Supported
H7	AIC → CLV	0.260	5.148***	Supported

### 4.3 Mediation analysis evaluation

Additionally, we found that B2B marketing processes significantly mediate the relationship between AIC and CLV. This confirms that AI-driven improvements in marketing information management, planning, and implementation lead to enhanced customer value, as firms can engage in more personalized and data-driven interactions with their customers (Sben et al., 2024). It has been observed that effective INFM has a significant and positive mediating effect on AIC and CLV relationships (*β* = 0.067, *t* = 3.56, *p* < 0.000), as well as IMPL (*β* = 0.091, *t* = 3.371, *p* < 0.001). However, the PLAN did not have a significant mediating impact on the AIC and CLV relationship (*β* = 0.062, *t* = 1.536, *p* = 0.125). Table 7 presents the results of the mediating effects.

**Table 7:** Results of indirect relationships.

Hypotheses	Path co-efficient	*t*-value	*p*-values	Decision
AIC→PLAN→CLV	0.062	1.536	0.125	N.S
AIC→INFM→CLV	0.067	3.56	0	Significant
AIC→IMPL→CLV	0.091	3.371	0.001	Significant

## 5 Discussion

The results of the direct and indirect relationships tested in this study provide insightful evidence of the role of AIC in enhancing B2B marketing processes and their impact on CLV. Through cutting-edge AI infrastructure, business-spanning strategies, and proactive adoption stances, firms can enhance their marketing capabilities and cultivate stronger customer relationships, ultimately leading to increased CLV. It also highlighted the importance of strategic investments in AI technologies and the expansion of marketing capabilities to harness the full potential of AI in B2B marketing. These findings are reliable with prior study that has emphasized the transformative role of AI in improving marketing decision-making processes and operational efficiencies (Dwivedi, 2024; Sben et al., 2024).

In terms of the relationships between marketing processes and CLV, the results indicate that both information management and implementation significantly influence CLV, supporting hypotheses H4 and H6. These findings align with existing literature suggesting that firms with robust marketing information management systems and effective marketing implementation strategies are better positioned to enhance customer relationships, thereby improving CLV (Firmansyah et al., 2024). However, the study found no support for the relationship between marketing planning and CLV, an unexpected result. One possible explanation for this could be the unique challenges faced by firms in emerging markets like Pakistan, where strategic marketing planning might be less effective in driving immediate customer outcomes due to market volatility, resource constraints, or the nascent stage of AI adoption (Han et al., 2021).

When examining the indirect effects of AIC on CLV through the mediating role of marketing processes, the results provide mixed support. The indirect effect of planning was not significant, which further reinforces the notion that marketing planning might not significantly mediate the relationship between AIC and CLV. However, the indirect effects of information management and implementation were both significant, suggesting that AIC can enhance CLV by improving firms’ ability to manage marketing information and execute marketing strategies effectively. These results align with the findings from (AboElHamd et al., 2020), who argue that AI-driven improvements in marketing operations lead to better customer insights and more effective customer engagement, ultimately increasing CLV.

The significance of AIC in driving both direct and indirect effects on CLV highlights the strategic importance of investing in AI infrastructure and adopting a proactive stance toward AI integration. Firms that leverage AI to expand the efficacy and effectiveness of their marketing processes are better equipped to create value for customers over the long term. As evidenced by prior studies, AI’s ability to enhance customer insights, automate routine tasks, and support personalized marketing interventions contributes to building stronger customer relationships, which are crucial for maximizing CLV (Curiskis et al., 2023; Berger and Nasr, 1998).

## 6 Theoretical contributions

This investigation adds to the theoretical structure by looking at how the RBV theory can be used to explain AIC and how they affect B2B marketing and CLV. The study supports RBV’s idea that companies can gain a competitive edge by using valuable, rare, unique, and unavoidable resources. It does this by thinking of AI infrastructure, business spanning, and a proactive stance as strategic resources. The findings demonstrate that AIC not only enhances internal marketing processes: such as information management and implementation but also drives long-term customer engagement and retention, which is critical for sustaining competitive advantage in B2B contexts. This extension of RBV in the AI-driven marketing realm adds a novel dimension to existing theoretical frameworks on resource deployment and strategic marketing.

Moreover, this investigation contributes to the expanding corpus containing data concerning the intermediary function of marketing strategies in the link between AIC and CLV. While prior research has explored the part of AI in improving marketing functions, this study provides a more sophisticated grasp of how specific AI interacts with marketing operations to enhance customer value. By testing the mediating effects of information management, planning, and implementation, this research overcomes a gap in the existing literature by providing empirical evidence. Support for the mechanisms through which AIC influence long-term customer outcomes. This contribution enriches both the AI and marketing literature by providing a comprehensive framework that links AIC with marketing effectiveness and customer-centric outcomes in B2B settings.

## 7 Practical implications

This study offers several practical implications for B2B firms seeking to enhance their marketing capabilities and drive CLV through AI integration. First, the findings suggest that investing in AI infrastructure, such as advanced data analytics and automation tools, can significantly improve key marketing processes, particularly in information management and implementation. B2B firms can leverage AI to gather deeper customer insights, automate routine tasks, and implement data-driven marketing strategies, thereby improving their ability to engage customers and foster long-term relationships. Companies that adopt a proactive stance toward AI and integrate it into their marketing operations can enhance both efficiency and effectiveness, leading to better customer outcomes and increased CLV.

Additionally, this study highlights the need for firms to focus on AI-driven marketing implementation rather than solely on strategic planning to improve CLV. While AI enhances marketing planning, its direct influence on CLV is more evident through the execution of marketing strategies. This suggests that businesses should prioritize the implementation of AI-driven initiatives that can directly affect customer interactions, such as personalized marketing campaigns and predictive analytics for customer retention. By focusing on AI’s role in improving real-time decision-making and execution, B2B firms can strengthen customer loyalty, drive higher lifetime value, and acquire a competitive advantage in their businesses.

These real-world implications provide B2B companies significant benefits in enhancing marketing capabilities and CLV through AI integration. Investing in automation and advanced analytics enables businesses to enhance customer insights, automate repetitive tasks, and optimize information management, thereby facilitating more precise and efficient customer engagement. Prioritizing AI-driven implementation over limited strategic planning enables businesses to focus on personalized, real-time interactions such as targeted marketing and predictive customer retention, thereby enhancing customer loyalty, increasing CLV, and providing a competitive advantage in an increasingly dynamic market.

## 8 Conclusion

This study demonstrates the significant role of AIC in enhancing B2B marketing processes and driving CLV, grounded in the RBV theory. By leveraging AI infrastructure, business spanning, and a proactive stance, firms can improve marketing effectiveness, particularly in information management and implementation, which in turn positively impacts CLV. However, the study also highlights that the relationship between AI-driven marketing planning and CLV might be less pronounced in certain contexts, particularly in developing markets. While the influence of AI on marketing planning is less clear, the findings offer valuable theoretical and practical insights, highlighting AI’s potential as a strategic asset for long-term customer engagement and competitive advantage. This research fills up gaps in the literature and provides actionable strategies for businesses seeking to harness AI to maximize customer value.

## 9 Limitation and future research

This study, whereas contributing beneficial insights, unveils several limitations that recommend exciting possibilities for future research. The study merely gathered data from Pakistani B2B firms, which might limit the generality of the findings to other nations as well as different sectors. Further investigations might widen the sample by incorporating organizations from various countries or industries consequently validating the results across various scenarios. Additionally, this investigation primarily focused on the role of AIC in marketing processes and CLV but did not explore potential moderating factors such as firm size, industry type, or market dynamics. Future work could investigate these variables to offer an additional perspective into the mechanisms of how AI impacts B2B marketing outcomes under varying conditions. We recommend executing longitudinal investigations to analyze the long-term impacts of AI integration on marketing performance and customer retention over time.

## Data Availability

The raw data supporting the conclusions of this article will be made available by the authors, without undue reservation.

## Appendix 1 Measurement constructs used in the study

**Table:**

Constructs	Source	Items
AIC INFS	Kumar et al. (1993); Mikalef et al. (2023)	1. Efficient data management services and robust architectures explicitly designed for AI applications. 2. Our AI infrastructure is designed to provide top-notch security for data, utilizing state-of-the-art technology to protect it from end to end.
BSAP	Kumar et al. (1993)	1. Establishing a well-defined understanding of how AI enhances business value. 2. Creating a highly efficient and adaptable AI planning process and crafting a strong AI plan.
PROS	Kumar et al. (1993)	1. We constantly explore and implement new AI tools and techniques to meet our evolving needs. 2. We foster an environment that encourages experimentation with AI. 3. We are constantly striving to find innovative methods to improve the efficiency of AI utilization. 4. We stay up-to-date with the latest advancements in AI technology.
B2B Marketing Capabilities INFM	Kumar et al. (1993); Mikalef et al. (2023)	1. Utilizing market research skills to create impactful marketing initiatives. 2. Monitoring customer preferences and requirements. 3. Utilizing marketing studies information to its fullest potential. 4. Examining our market data.
PLAN	Kumar et al. (1993)	1. Skilled in efficiently segmenting and targeting markets. 2. Effective marketing management abilities and procedures. 3. Creating innovative marketing plans. 4. Ensuring a comprehensive approach to marketing procedures for planning.
IMPL	Kumar et al. (1993)	1. Optimizing the allocation of marketing resources. 2. Efficiently coordinating the delivery of marketing strategies. 3. Implementing marketing strategies with precision and expertise.
CLV	Kumar and Venkatesan (2021)	1. Comprehensive Evaluation of Product/Service Satisfaction. 2. Strong inclination to promote ideas and initiatives among colleagues in the industry. 3. Perceived Economic Effectiveness of Offerings. Chances of being supported by professional affiliates.
